# Comparison of walking overground and in a Computer Assisted Rehabilitation Environment (CAREN) in individuals with and without transtibial amputation

**DOI:** 10.1186/1743-0003-9-81

**Published:** 2012-11-14

**Authors:** Deanna H Gates, Benjamin J Darter, Jonathan B Dingwell, Jason M Wilken

**Affiliations:** 1 Department of Orthopaedics & Rehabilitation, Center for the Intrepid, Brooke Army Medical Center, Ft. Sam Houston, TX, 78234, USA; 2 Department of Physical Therapy, Virginia Commonwealth University, Richmond, VA, 23298, USA; 3 Department of Kinesiology & Health Education, University of Texas, Austin, TX, 78712, USA

**Keywords:** Treadmill, Kinematics, Virtual reality, Rehabilitation

## Abstract

**Background:**

Due to increased interest in treadmill gait training, recent research has focused on the similarities and differences between treadmill and overground walking. Most of these studies have tested healthy, young subjects rather than impaired populations that might benefit from such training. These studies also do not include optic flow, which may change how the individuals integrate sensory information when walking on a treadmill. This study compared overground walking to treadmill walking in a computer assisted virtual reality environment (CAREN) in individuals with and without transtibial amputations (TTA).

**Methods:**

Seven individuals with traumatic TTA and 27 unimpaired controls participated. Subjects walked overground and on a treadmill in a CAREN at a normalized speed. The CAREN applied optic flow at the same speed that the subject walked. Temporal-spatial parameters, full body kinematics, and kinematic variability were collected during all trials.

**Results:**

Both subject groups decreased step time and control subjects decreased step length when walking in the CAREN. Differences in lower extremity kinematics were small (< 2.5^○^) and did not exceed the minimal detectable change values for these measures. Control subjects exhibited decreased transverse and frontal plane range of motion of the pelvis and trunk when walking in the CAREN, while patients with TTA did not. Both groups exhibited increased step width variability during treadmill walking in the CAREN, but only minor changes in kinematic variability.

**Conclusions:**

The results of this study suggest that treadmill training in a virtual environment should be similar enough to overground that changes should carry over. Caution should be made when comparing step width variability and step time results from studies utilizing a treadmill to those overground.

## Background

Treadmills have many advantages over typical overground labs for gait training. For one,they allow for continuous collection of data within a small capture volume. They can also be integrated with virtual reality systems to provide visual cues, including optic flow and real-time feedback. Treadmills can be useful for both assessment of gait and fall risk and potentially for gait retraining [1]. For treadmill based training to improve real-world function, treadmill and overground gait must have similar underlying processes such that practice on the treadmill can effectively transfer to performance overground [2]. While the task of walking should, in theory, be mechanically equivalent on treadmills and overground [3], some data suggests that there are differences, including altered kinematics [4,5], kinetics [5,6], and energy costs [7,8]. This is particularly common in populations other than young healthy adults, such as patients with hemiplegia [9,10] and healthy elderly [11-13].

Previous researchers have speculated that differences might be caused by various factors including: differences in compliance of the walking surface [14], subtle intra-stride variations in treadmill belt speed [15], the constraint of treadmill (narrow belts and railings), and unfamiliarity of walking on a treadmill [16]. Others suggested that differences may arise from the altered sensory feedback encountered on a treadmill. In particular, visual feedback is incongruous since a visual sense of movement caused by the relative motion between an observer and the environment (optic flow) is lacking [3,17]. Experimental variations in visual flow in healthy adults caused modulations in walking speed [18-20], stride length [19,21], and cadence [20,21]. Sheik-Nainar and Kaber studied the gait pattern of individuals during treadmill walking with and without a virtual reality (VR) system providing optic flow [20]. In their study, treadmill walking lead to a flatter foot contact angle and decreased knee flexion compared to overground walking. When optic flow was added during treadmill walking, the knee flexion angle was indistinguishable from overground walking [20].

One population that may benefit from treadmill training in a virtual environment is individuals with amputation [1]. The assumption of equivalence between treadmill and overground walking may, however, be questionable in this population. Persons with transtibial amputation (TTA) may have difficulty adjusting to the altered constraints of treadmill locomotion [22,23]. They may also be more reliant on visual information than able-bodied adults due to the loss of proprioception from their involved limb [24,25]. For patients with transtibial or transfemoral amputations, walking on the treadmill was about two and a half times more energetically costly than walking overground [8]. Only one case-series has examined the kinematic differences between overground and treadmill walking without the use of virtual reality in persons with TTA [23]. For two of the three participants in that study, the asymmetry of stride and stance times were reduced on the treadmill compared to overground [23]. Small differences in peak angles at the hip and knee between overground and treadmill walking (< 2.8°) were also reported but not compared statistically, due to the small sample size.

Overall, the changes that have been reported between the two walking conditions have been small. As such, even the differences that reach statistical significance may not be physically or functionally meaningful. Riley et al. measured the coefficient of repeatability for healthy, able-bodied individuals walking overground to determine if differences observed between overground and treadmill walking were greater than expected measurement variability [5]. The coefficient of repeatability is a measure of precision which is conceptually similar to the minimal detectable change value [26] that indicates the minimum level of change required to have 95% confidence that a real change occurred between conditions. Riley et al. found that, in all cases, the mean kinematic differences between overground and treadmill were less than this coefficient. The only difference in kinetics that exceeded it were knee extension moment, and anterior-posterior maximum and medial-lateral minimum ground reaction forces [5].

The purpose of this study was to determine if there were kinematic and/or temporal-spatial differences between overground walking and treadmill walking in a computer assisted rehabilitation environment (CAREN) in healthy control subjects or in individuals with TTA. This system has the advantage of complete immersion in an environment where the visual scene moves at the speed the subject is walking, providing appropriate optic flow. The system also consists of a wide treadmill belt to minimize changes that might occur due to any width constraint of the treadmill. We hypothesized that there would be no temporal-spatial or kinematic differences between overground walking and treadmill walking in a virtual environment for either group.

## Methods

### Subjects

Seven healthy young men with traumatic transtibial amputations (TTA) participated (Table 1). All participants were screened to ensure that they were able to independently ambulate without an assistive device, for at least five minutes at a time, for a minimum of two months prior to testing. Subjects were also screened to ensure they had a healthy contralateral limb and pain levels of less than 4 of 10 on a subjective pain scale (0 = no pain, 10 = maximum pain) for their involved limb. Patients were excluded if they had any open wounds on their residual limb. Data were also collected from a control group consisting of 27 (22 male, 5 female) healthy, young adults (Table 1). The research protocol was approved by Brooke Army Medical Center’s Institutional Review Board and all participants gave their written informed consent prior to participation.

**Table 1:** Characteristics of subjects with traumatic transtibial amputation (TTA) and unimpaired controls

**Subjects with TTA (n = 7)**	**Subject**	**Age (years)**	**Height (m)**	**Mass (kg)**	**Leg Length (m)**	**Time since amputation**	**Affected Limb**	**Prosthesis Type**
	S01	33	1.88	117.9	0.93	3 yrs	L	Renegade LP ^a^
	S02	23	1.86	86.8	0.95	4 mos	R	Vari-flex ^b^
	S03	21	1.78	96.6	0.93	7 mos	L	Renegade LP ^a^
	S04	32	1.93	102.0	0.92	2 yrs	R	Proprio^b^
	S05	28	1.80	97.0	0.95	10 mos	R	LP Vari-flex^b^
	S06	38	1.84	98.9	0.90	1 yr	L	Vari-flex^b^
	S07	40	1.73	95.6	0.94	6 mos	R	Vari-flex^b^
Mean (SD)		30 (7)	1.85 (0.06)	99.2 (9.5)	0.97 (0.05)	1.2 (1) yrs	3 L/4 R	
Controls (n = 27)	Mean (SD)	23 (6)	1.71 (0.13)	75.7 (11.7)	0.91 (0.07)			

^a^ Manufactured by Freedom Innovations, LLC (Irvine, CA, USA).

^b^ Manufactured by Össur (Reyjavik, Iceland).

### Experimental protocol

All subjects participated in a biomechanical gait assessment, first during overground and then during treadmill walking. Full body kinematics were captured using a six-degrees of freedom marker set [27] according to [26]. Additionally, the locations of 20 bony landmarks in relation to marker clusters were found by manual palpation and recorded using a digitizing pointer (C-Motion, Inc., Germantown, MD). Walking speed was non-dimensionally scaled to each subject’s leg length, *l*, according to $Walking\;Speed\;=\;\sqrt{Fn\;\cdot \;g\;\cdot \;l}$, where g is the gravitational constant, Fn is the Froude Number [28]. Subjects walked at Fn = 0.16, which corresponded to an average speed of 1.19 m/s for control subjects and 1.23 m/s for TTA. Twenty (10 left and 10 right) strides were collected for each subject during overground and treadmill walking.

For all overground walking trials, subjects walked on a 10-m walkway while kinematics were collected at 120 Hz using a 26-camera motion capture system (Motion Analysis, Santa Rosa, CA). An audible cuing system provided real-time feedback of walking speed by generating a continuous tone when the subject’s speed was within the prescribed range (± 5% target speed). If an inconsistent tone or no tone was generated, subjects were instructed to walk faster or slower until the tone sounded. Strides were only chosen from trials where the tone was heard consistently. Due to limits of the capture volume, the 20 strides collecting during overground walking were not contiguous.

**Figure 1:**
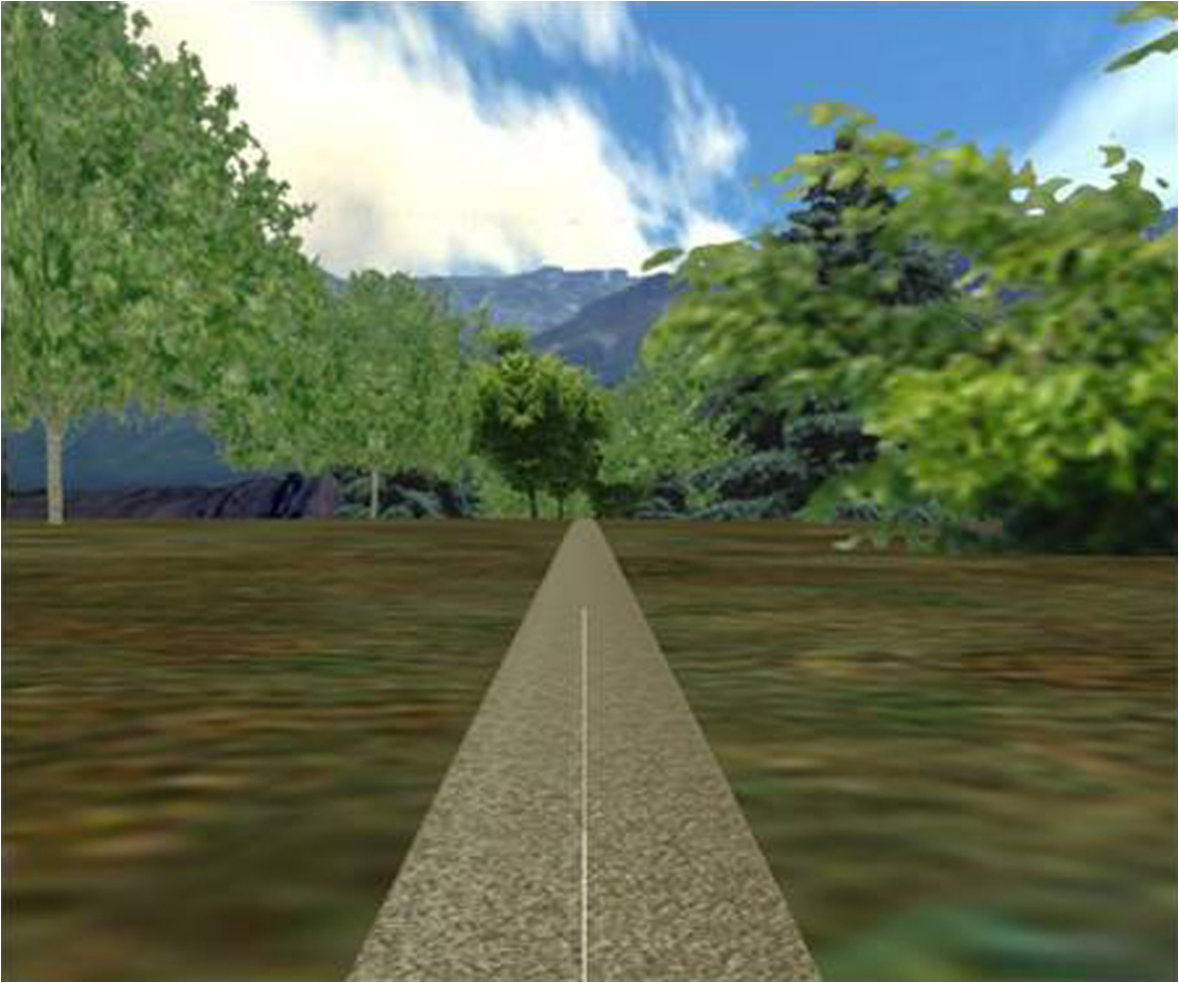
**The Computer Assisted Rehabilitation Environment (CAREN).** Picture depicting the virtual environment that was projected around the subjects as they walked on the treadmill.

During treadmill walking, subjects walked in a Computer Assisted Rehabilitation Environment (CAREN) system (Motek, Amsterdam, Netherlands) consisting of a 7-m diameter dome with a virtual environment projected 300° around the subject [29]. Subjects walked on a 2 m wide by 3 m long single-belt treadmill embedded in a 4 m diameter platform that was flush with the treadmill belt. The virtual reality scene used during the treadmill walking depicted a path through a forest (Figure 1). Subjects were instructed to focus their gaze on the end of the path and walk straight ahead along it. Subjects were monitored to ensure they kept their heads facing forward. After a two and a half minute familiarization period, kinematic data were collected at 60 Hz using a 24-camera Vicon motion capture system (Vicon, Oxford, UK). All subjects in both groups had previous experience with treadmill walking and all wore their own athletic shoes during both overground and treadmill walking. Two of the subjects with TTA (S06 and S07) had prior experience walking in the CAREN system.

### Data analysis

Marker position data were filtered using a 4^th^ order low-pass Butterworth filter with a 6 Hz cut-off frequency. Marker positions and landmarks were used to create a 13 segment whole-body model using Visual 3D software (C-Motion, Inc., Germantown, MD) consisting of feet, shanks, thighs, arms, forearms, pelvis, trunk and head. Local coordinate systems for the segments were defined according to the International Society of Biomechanics’ recommendations [30,31]. Angular motion of the ankle, knee, and hip were defined using Euler angles according to rotation sequences recommended by [30,31]. Joint kinematic data were resampled such that there were exactly 101 points per gait cycle. Peak joint kinematics were determined by taking the maximum value attained between 0 and 25% of the gait cycle (early stance), 25 and 65% (late stance) and 65 and 100% (swing). Range of motion was calculated as difference between the maximum and minimum angles achieved at any point in the gait cycle.

Heel strikes were determined using a velocity-based detection algorithm [32] and then verified by visual inspection. Step length and step width were defined as the distance between the right and left heel markers at heel strike in the anterior-posterior and medial-lateral directions, respectively. Step time was the time elapsed between subsequent right and left heel strikes. For temporal-spatial measures of step time, step length, and step width, the standard deviation across all 10 cycles collected for each limb and condition represented the within-subject variability of that measure. For joint kinematics, variability was quantified as MeanSD: the average width of the standard deviation across the movement cycle [33].

### Statistical analysis

As control and TTA subjects were not height, weight or age matched, comparisons were only made within the groups and not between them. Separate two-factor within-subjects ANOVAs were used for each dependent measure, in each test group, to test for differences between walking environments (CAREN, Overground) and limbs (right, left and prosthetic, intact) (SPSS 16, Chicago, IL). Significant Limb × Condition interaction effects were explored using the estimated marginal means with a Bonferroni correction. P-values are denoted ‘Cond’ and ‘Limb’ for condition and limb effects, respectively. To assist in the clinical interpretation, significant differences in peak joint angles were then compared to minimal detectable change (MDC) values for this marker set given in [26,34]. MDC is the amount of change which is sufficiently greater than measurement error to indicate a true change has occurred in the variable of interest [35]. Thus observed differences between treadmill walking in the CAREN and overground walking were only considered true differences if they exceeded the MDC.

## Results

### Temporal-spatial step measures

**Figure 2:**
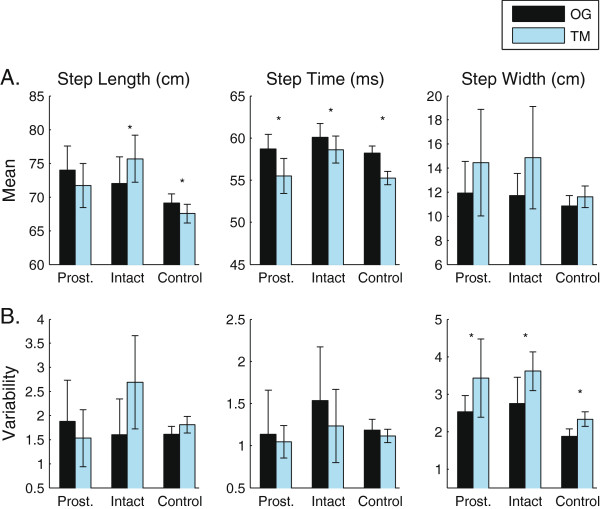
**Temporal-spatial parameters during overground and treadmill walking.A**) Means and **B**) Variability of temporal-spatial measures are shown for subjects walking overground (OG) and on a treadmill (TM) with optic flow. Errorbars represent the 95% confidence intervals about the means. Significant differences (p < 0.05) in peak angles between OG and TM are highlighted by ‘*’.

Control subjects decreased step length (p_Cond_ = 0.005) and step time (p_Cond_ < 0.001) on the treadmill compared to overground (Figure 2A; Additional file 1: Table S1). They also exhibited asymmetry in step width (Right: 11.4 (3.2) cm; Left: 11.1 (3.3) cm; p_Limb_ < 0.005). Subjects with transtibial amputations (TTA) took longer steps on their intact limb when walking on the treadmill than walking overground (p_Limb×Cond_ = 0.016). Their intact limb step length increased from 72.0 cm to 75.6 cm (p = 0.028; Figure 2), while their prosthetic limb step length decreased slightly (74.0 to 71.7 cm; p = 0.114). They also decreased their step time on both limbs when walking on the treadmill (p_Cond_ = 0.032; Additional file 1). The difference between the conditions was greater on the prosthetic side (p_Limb_ = 0.046, p_Limb×Cond_ = 0.035). There were no changes in step width between the two conditions, although there was a tendency for both control and TTA subjects to increase step width on the treadmill (Controls: p_Cond_ = 0.082, TTA: p_Cond_ = 0.110). Additionally, both subjects with TTA (p_Cond_ = 0.029) and controls (p_Cond_ = 0.004) exhibited increased step width variability on the treadmill compared to overground (Figure 2B; Additional file 1: Table S1).

### Joint kinematics

**Figure 3:**
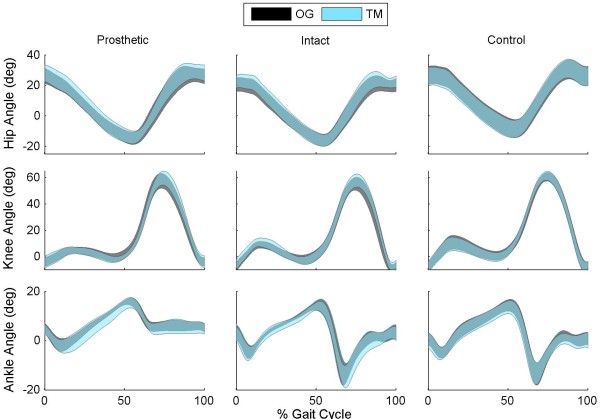
**Sagittal plane joint angles during overground and treadmill walking.** Ankle, knee, and hip angles are shown for subjects walking overground (OG) and walking on a treadmill (TM) with optic flow. Data are shown for the intact and prosthetic limb of the patients with transtibial amputation and for the average of the right and left limb of control subjects. Peak joint angles were measured during early stance (0-20% gait cycle), late stance (25-65% gait cycle), and swing (65-100% gait cycle). Significant differences (p < 0.05) in peak angles between OG and TM are highlighted by ‘*’.

Control subjects exhibited increased ankle dorsiflexion during swing, and increased knee flexion during early stance, late stance, and swing when walking on overground compared to walking on the treadmill (p_Cond_ ≤ 0.043; Figure 3). While statistically significant, these differences were all less than 1.2^○^, which were less than the MDC values for those measures (Additional file 1: Table S2). Subjects with TTA walked with increased knee flexion on their intact limb during early stance on the treadmill (p_Limb_ = 0.001; p_Limb×Cond_ = 0.081). They also increased knee extension 1.3^○^ during late stance (p_Cond_ = 0.025; Figure 3) and increased knee flexion 2.4^o^ during swing when walking on the treadmill (p_Cond_ = 0.042). All of these differences were also less than the MDC values (Additional file 1: Table S2).

**Figure 4:**
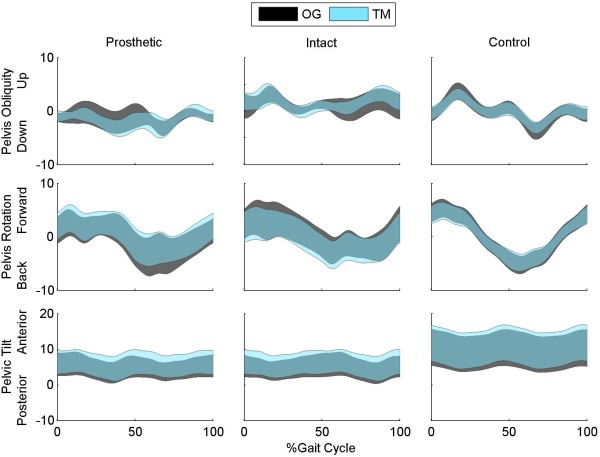
**Three dimensional pelvic motion during overground and treadmill walking.** Pelvic obliquity, rotation and tilt angles are shown for subjects walking overground (OG) and walking on a treadmill (TM) with optic flow. Data are shown for the intact and prosthetic limb of the patients with transtibial amputation and for the average of the right and left limb of control subjects.

**Figure 5:**
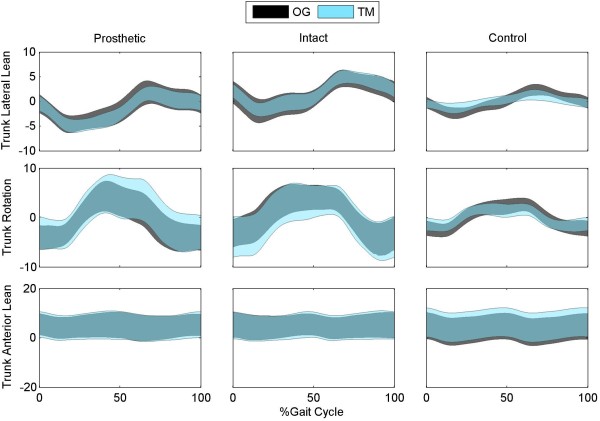
**Three-dimensional trunk motion during overground and treadmill walking.** Trunk lateral lean, rotation, and anterior lean angles are shown for subjects walking overground (OG) and walking on a treadmill (TM) with optic flow. Data are shown for the intact and prosthetic limb of the patients with transtibial amputation and for the average of the right and left limb of control subjects.

For the control subjects, there was a significant decrease in range of motion for the pelvic obliquity angle, pelvic rotation, trunk lateral lean angle and trunk rotation angles on the treadmill compared to overground (p_Cond_ ≤ 0.004; Figures 4 and 5). In contrast, there were no significant differences in pelvis or trunk range of motion between walking condition in any plane (p_Cond_ > 0.097) for the subjects with TTA. There was a significant increase in pelvic obliquity range of motion during strides on the prosthetic side compared to intact side (p_L_ = 0.049).

### Kinematic variability

**Figure 6:**
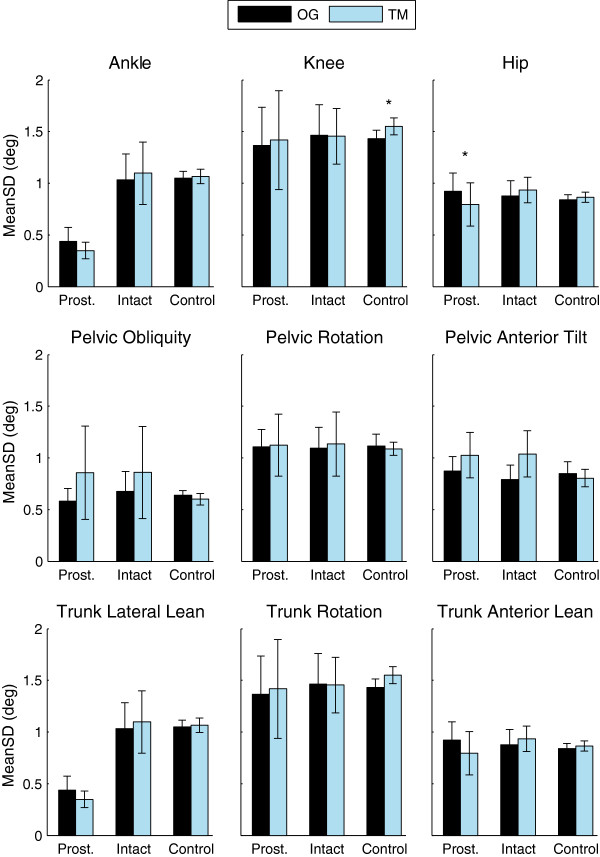
**Variability of Joint Angles during overground and treadmill walking.** The MeanSD of ankle, knee, and hip angles in the sagittal plane, and all three planes for the pelvis and trunk are shown for subjects walking overground (OG) and walking on a treadmill (TM) with optic flow. Errorbars represent the 95% confidence intervals about the means. Significant differences (p < 0.05) in peak angles between OG and TM are highlighted by ‘*’.

Kinematic variability, MeanSD, was not different between overground and treadmill walking at the ankle or hip for the control subjects (p > 0.49; Figure 6). MeanSD of the knee was greater on the treadmill than overground (p = 0.031). Subjects with TTA exhibited decreased MeanSD of the ankle on their prosthetic limb compared to their intact limb (Figure 6; p_Limb_ < 0.001). There was no difference in MeanSD of the ankle or knee between the two conditions for either limb (p_Cond_ > 0.866). MeanSD of the hip decreased on the treadmill for the prosthetic side only (p_Limb×Cond_ = 0.036; Figure 6; Additional file 1: Table S3).

## Discussion

This study compared overground and treadmill walking in individuals with transtibial amputations (TTA) and healthy controls. While numerous studies have compared these two walking conditions, this is the first study to include a wide treadmill surface surrounded by a virtual environment (CAREN) that applied optic flow at the same rate the subject walked. This environment might encourage a walking pattern that is more similar to overground walking than has previously been found. Alternatively, this may not help normalize conditions if the chief contributing factors to the differences are related to subtle variations in treadmill belt speed, the increased compliance of the treadmill belt surface, altered vestibular inputs, or other factors. We found that both groups walked with similar overall kinematics and kinematic variability on the treadmill as they did overground (Figure 3). While there were several differences in lower extremity joint kinematics that reached statistical significance, all were less than the minimal detectable change, MDC, values [26,34].

### Temporal-spatial measures

The observed changes in temporal spatial measures agree with previous findings in healthy young adults. Both groups tested here exhibited decreased step time when walking on the treadmill compared to overground (Figure 2). Similarly, previous studies found that healthy young adults increase their cadence when walking on a treadmill in the absence of optic flow [4,7,17]. Also, similar to previous work, control subjects reduced their step length when walking on the treadmill [7,17]. In contrast, patients with TTA significantly increased their step length on their intact limb (Figure 2), while slightly decreasing it on their prosthetic side. Control subjects exhibited a significant asymmetry in step width, but no other measures. One limitation is that we did not collect data on limb dominance, and it is possible that additional asymmetries may have emerged if the dominant limb were compared to the non-dominant limb rather than right to left [36].

Previously, healthy young adults increased step width [37,38] but decreased step width variability [38] when walking on a treadmill compared to overground. One possible reason for this change is that subjects increased step width to increase or maintain lateral stability because of a greater perceived risk associated with errors in foot placement (ie. stepping off the treadmill) [38]. In the present study there was a tendency for both control subjects and patients with TTA to increase their step width when walking on the treadmill, but this did not reach statistical significance for either group. The aforementioned studies tested subjects on a 0.457 m wide treadmill belt that was raised from the floor [37,38]. In the current study, subjects walked on a wide treadmill belt that was flush with the surrounding surface. Similar to walking on a sidewalk, subjects could freely drift from side to side on the treadmill, without fear of stepping off the side of the treadmill belt. When walking on the treadmill in the CAREN, subjects wore a harness that attached to a support structure out of the subjects field of view (See [29] for image). This may have led to an increased feeling of safety. The harness was adjusted to allow free movement within the constraints of the belt, but in the rare event that they approached the outer limits of the treadmill, they would feel a pull on the rope. Thus the subjects may not have perceived the same increased risk associated with errors in foot placement that they would have on a narrow treadmill belt without a harness.

In contrast to previous findings [37,38], both subject groups exhibited *increased* step width variability when walking on the treadmill in the CAREN. The decreased constraints of the treadmill as compared to previous studies may also have contributed to this finding. Traditional treadmills entrain individuals to walk at a constant speed in a relatively narrow, straight path [14]. As mentioned above, the narrow width does not allow subjects to drift as much as they can on the treadmill in the CAREN. This narrow width may alter the step-to-step dynamics as subjects have to actively control their step width and step length to stay centered on the treadmill. This is not necessary in the CAREN, since there is a much wider space for the subjects to move in. It is also possible that the differences in step width variability are an artifact of subject drift. The calculations of step width do not account for changes in heading direction which may have occurred on a stride-to-stride basis.

### Kinematics

The small, but significant, differences in lower extremity kinematics between overground and treadmill walking agree with previous findings in healthy, young adults [4,5,16]. This is the first study to report trunk and pelvis motion during the two tasks and the first to statistically compare the kinematics of patients with TTA. Control subjects exhibited significant decreases in pelvis and trunk range of motion in the frontal and transverse planes during treadmill walking in the CAREN (Figure 4,5). All of these differences exceeded the MDC values, except for trunk lateral lean range of motion. This finding may be a product of altered gait associated with walking on the treadmill or simply a product of the small MDC values for these parameters. Another possible reason is the use of a harness which may restrict trunk motion. However, if the differences were purely related to the harness, we would have expected to see similar decreases in range of motion for the patients with TTA. As there were no differences in the patient group (p > 0.438), it is unlikely the differences are purely related to the harness.

Interestingly, even in persons with TTA, there were no differences in the kinematics between conditions that exceeded the MDC values. Yet, a previous study found that persons with TTA can exhibit large increases in energy costs when walking on the treadmill compared to overground (~2.5 × greater) [8] suggesting significantly altered gait mechanics. However, the patients were older (mean age: 56), had been in their prostheses only a short time (~2 months), and a majority of the subjects used walking aids such as canes, crutches, or walkers. Therefore, those results may reflect a novice and potentially more impaired population. As a result, the minimal differences in kinematics between overground and treadmill walking in this patient group might not be generalizable to an older, less fit population.

### Kinematic variability

Few studies have looked at the effects of treadmill walking on the variability of kinematics. Dingwell et al. studied healthy young adults and found significantly reduced variability of sagittal plane ankle angles and a trend toward decreased variability at the knee and hip during treadmill walking [14]. They also found that MeanSD of the upper body accelerations decreased on the treadmill compared to overground [14]. Conversely, a similar study by Terrier and Dériaz found no differences between conditions [39]. Here we saw two statistically significant differences between overground and treadmill walking in the CAREN. For subjects with TTA, there was a decrease in variability of the sagittal plane hip angle on the prosthetic side when walking on the treadmill, while in control subjects there was an increase in variability of the knee angle (Figure 4). In both cases, the average difference in MeanSD was less than a half degree (Additional file 1: Table S3). Thus there is minimal difference in kinematic variability between overground and treadmill walking in a virtual environment.

### Optic flow

If the subtle differences in kinematics between treadmill and overground walking were purely due to the lack of optic flow during treadmill walking, then, in theory, we would expect no difference between overground walking and treadmill walking in the CAREN. Instead we showed that the small differences in temporal spatial parameters and kinematics remained, and were comparable to those found in previous studies. There may be several reasons for this. First, there is a potential sensory mismatch between the visual inputs, that indicate forward motion, and the vestibular inputs, which do not. There is also some difference between how the visual field moves when walking overground versus walking in the CAREN. The scene is artificial and the graphics do not quite approach the level of realism. Additionally, despite drawing the treadmill through the virtual environment, the person’s perception of self motion may not match that of overground walking [40]. Locomotion may still be controlled with respect to the treadmill, not the passing world (ie. they are moving forward in the environment but are not moving with respect to the treadmill) [40].

## Conclusions

This study compared overground walking to walking on a treadmill in a virtual environment in both healthy adults and patients with transtibial amputations. While statistically significant differences in joint kinematics were observed in both groups, they were small and rarely greater than measurement error. These findings demonstrate the similarity of treadmill and overground gait and support the continued use of virtual reality based treadmill training aimed at improving overground walking. Mean step length and mean step time, as well as step width variability differed significantly between conditions and thus caution should be used when making comparisons of these measures between studies utilizing a treadmill and those where subjects walked overground.

## Competing interests

The authors declare that they have no competing interests.

## Authors’ contributions

DG was involved in the analysis and interpretation of the data, as well as drafting the manuscript. BD participated in the design of the study, data collection, and critically revised the manuscript for its intellectual content. JD and JW participated in the design of the study and critically revised the manuscript for its intellectual content. All authors have read and approved the final manuscript.

## Supplementary Material

Additional file 1 **Table S1.** Mean and standard deviation of the temporal-spatial parameters during overground (OG) and treadmill walking in a CAREN (CA). Significant differences between conditions are highlighted. Significant p-values (p < 0.05) are in bold. Click here for file

## References

[B1] Darter BJ Wilken JM Gait training with virtual reality-based real-time feedback: improving gait performance following transfemoral amputation Phys Ther 2011 91 9 1385 1394 10.2522/ptj.20100360 21757579

[B2] Schmidt RA Lee T Motor Control and Learning: A Behavioral Emphasis 2011 5th Champaign, IL: Human Kinetics

[B3] van Ingen Schenau GJ Some fundamental aspects of the biomechanics of overground versus treadmill locomotion Med Sci Sports Exerc 1980 12 4 257 261 7421475

[B4] Strathy GM Chao EY Laughman RK Changes in knee function associated with treadmill ambulation J Biomech 1983 16 7 517 522 10.1016/0021-9290(83)90066-0 6619169

[B5] Riley PO Paolini G Della U Croce K Paylo W Kerrigan DC A kinematic and kinetic comparison of overground and treadmill walking in healthy subjects Gait Posture 2007 26 1 17 24 10.1016/j.gaitpost.2006.07.003 16905322

[B6] White SC Yack HJ Tucker CA Lin HY Comparison of vertical ground reaction forces during overground and treadmill walking Med Sci Sports Exerc 1998 30 10 1537 1542 10.1097/00005768-199810000-00011 9789855

[B7] Pearce ME Cunningham DA Donner AP Rechnitzer PA Fullerton GM Howard JH Energy cost of treadmill and floor walking at self-selected paces Eur J Appl Physiol Occup Physiol 1983 52 1 115 119 10.1007/BF00429037 6686120

[B8] Traballesi M Porcacchia P Averna T Brunelli S Energy cost of walking measurements in subjects with lower limb amputations: a comparison study between floor and treadmill test Gait Posture 2008 27 1 70 75 10.1016/j.gaitpost.2007.01.006 17360186

[B9] Harris-Love ML Forrester LW Macko RF Silver KH Smith GV Hemiparetic gait parameters in overground versus treadmill walking Neurorehabil Neural Repair 2001 15 2 105 112 10.1177/154596830101500204 11811252

[B10] Harris-Love ML Macko RF Whitall J Forrester LW Improved hemiparetic muscle activation in treadmill versus overground walking Neurorehabil Neural Repair 2004 18 3 154 160 10.1177/0888439004267678 15375275

[B11] Greig C Butler F Skelton D Mahmud S Young A Treadmill walking in old age may not reproduce the real life situation J Am Geriatr Soc 1993 41 1 15 18 8418117 10.1111/j.1532-5415.1993.tb05941.x

[B12] Wass E Taylor NF Matsas A Familiarisation to treadmill walking in unimpaired older people Gait Posture 2005 21 1 72 79 10.1016/j.gaitpost.2004.01.003 15536036

[B13] Parvataneni K Ploeg L Olney SJ Brouwer B Kinematic, kinetic and metabolic parameters of treadmill versus overground walking in healthy older adults Clin Biomech 2009 24 1 95 100 10.1016/j.clinbiomech.2008.07.002 18976839

[B14] Dingwell JB Cusumano JP Cavanagh PR Sternad D Local dynamic stability versus kinematic variability of continuous overground and treadmill walking J Biomech Eng 2001 123 1 27 32 10.1115/1.1336798 11277298

[B15] Savelberg HH Vorstenbosch MA Kamman EH van de Weijer JG Schambardt HC Intra-stride belt-speed variation affects treadmill locomotion Gait Posture 1998 7 1 26 34 10.1016/S0966-6362(97)00023-4 10200372

[B16] Matsas A Taylor N McBurney H Knee joint kinematics from familiarised treadmill walking can be generalised to overground walking in young unimpaired subjects Gait Posture 2000 11 1 46 53 10.1016/S0966-6362(99)00048-X 10664485

[B17] Arsenault AB Winter DA Marteniuk RG Treadmill versus walkway locomotion in humans: an EMG study Ergon 1986 29 5 665 676 10.1080/00140138608968301 3720723

[B18] Lamontagne A Fung J McFadyen BJ Faubert J Modulation of walking speed by changing optic flow in persons with stroke J Neuroeng Rehabil 2007 4 22 10.1186/1743-0003-4-22 17594501 PMC1913055

[B19] Prokop T Schubert M Berger W Visual influence on human locomotion. Modulation to changes in optic flow Exp Brain Res 1997 114 1 63 70 10.1007/PL00005624 9125452

[B20] Sheik-Nainar MA Kaber DB The utility of a virtual reality locomotion interface for studying gait behavior Hum Factors 2007 49 4 696 709 10.1518/001872007X215773 17702221

[B21] Pailhous J Ferrandez AM Fluckiger M Baumberger B Unintentional modulations of human gait by optical flow Behav Brain Res 1990 38 3 275 281 10.1016/0166-4328(90)90181-D 2363843

[B22] Waters RL Mulroy S The energy expenditure of normal and pathologic gait Gait Posture 1999 9 3 207 231 10.1016/S0966-6362(99)00009-0 10575082

[B23] Button C Moyle S Davids K Comparison of below-knee amputee gait performed overground and on a motorized treadmill Adapt Phys Activ Q 2010 27 2 96 112 20440022 10.1123/apaq.27.2.96

[B24] Vanicek N Strike SC McNaughton L Polman R Lower limb kinematic and kinetic differences between transtibial amputee fallers and non-fallers Prosthet Orthot Int 2010 34 4 399 410 10.3109/03093646.2010.480964 20450461

[B25] Fernie GR Holliday PJ Postural sway in amputees and normal subjects J Bone Joint Surg Am 1978 60 7 895 898 701337

[B26] Wilken JM Rodriguez KM Brawner M Darter BJ Reliability and minimal detectible change values for gait kinematics and kinetics in healthy adults Gait Posture 2012 35 2 301 307 10.1016/j.gaitpost.2011.09.105 22041096

[B27] Collins TD Ghoussayni SN Ewins DJ Kent JA A six degrees-of-freedom marker set for gait analysis: repeatability and comparison with a modified Helen Hayes set Gait Posture 2009 30 2 173 180 10.1016/j.gaitpost.2009.04.004 19473844

[B28] Vaughan CL O'Malley MJ Froude and the contribution of naval architecture to our understanding of bipedal locomotion Gait Posture 2005 21 3 350 362 10.1016/j.gaitpost.2004.01.011 15760752

[B29] McAndrew PM Dingwell JB Wilken JM Walking variability during continuous pseudo-random oscillations of the support surface and visual field J Biomech 2010 43 8 1470 1475 10.1016/j.jbiomech.2010.02.003 20346453 PMC2866814

[B30] Wu G van der Helm FC Veeger HE Makhsous M Van Roy P Anglin C Nagels J Karduna AR McQuade K Wang X Werner FW Buchholz B ISB recommendation on definitions of joint coordinate systems of various joints for the reporting of human joint motion–Part II: shoulder, elbow, wrist and hand J Biomech 2005 38 5 981 992 10.1016/j.jbiomech.2004.05.042 15844264

[B31] Wu G Siegler S Allard P Kirtley C Leardini A Rosenbaum D Whittle M D'Lima DD Cristofolini L Witte H Schmid O Stokes I ISB recommendation on definitions of joint coordinate system of various joints for the reporting of human joint motion–part I: ankle, hip, and spine J Biomech 2002 35 4 543 548 10.1016/S0021-9290(01)00222-6 11934426

[B32] Zeni JA Jr Richards JG Higginson JS Two simple methods for determining gait events during treadmill and overground walking using kinematic data Gait Posture 2008 27 4 710 714 10.1016/j.gaitpost.2007.07.007 17723303 PMC2384115

[B33] Dingwell JB Marin LC Kinematic variability and local dynamic stability of upper body motions when walking at different speeds J Biomech 2006 39 3 444 452 10.1016/j.jbiomech.2004.12.014 16389084

[B34] Wilken J Rodriguez K Brawner M Darter B Reliability and minimal detectible change values for gait kinematics and kinetics in healthy adults Gait Posture In Press 10.1016/j.gaitpost.2011.09.105 22041096

[B35] de Vet HC Terwee CB Ostelo RW Beckerman H Knol DL Bouter LM Minimal changes in health status questionnaires: distinction between minimally detectable change and minimally important change Health Qual Life Outcomes 2006 4 54 10.1186/1477-7525-4-54 16925807 PMC1560110

[B36] Sadeghi H Allard P Prince F Labelle H Symmetry and limb dominance in able-bodied gait: a review Gait Posture 2000 12 1 34 45 10.1016/S0966-6362(00)00070-9 10996295

[B37] Stolze H Kuhtz-Buschbeck JP Mondwurf C Boczek-Funcke A Johnk K Deuschl G Illert M Gait analysis during treadmill and overground locomotion in children and adults Electroencephalogr Clin Neurophysiol 1997 105 6 490 497 10.1016/S0924-980X(97)00055-6 9448652

[B38] Rosenblatt NJ Grabiner MD Measures of frontal plane stability during treadmill and overground walking Gait Posture 2010 31 3 380 384 10.1016/j.gaitpost.2010.01.002 20129786

[B39] Terrier P Deriaz O Kinematic variability, fractal dynamics and local dynamic stability of treadmill walking J Neuroeng Rehabil 2011 8 12 10.1186/1743-0003-8-12 21345241 PMC3060113

[B40] Durgin FH Pelah A Fox LF Lewis J Kane R Walley KA Self-motion perception during locomotor recalibration: more than meets the eye J Exp Psychol Hum Percept Perform 2005 31 3 398 419 15982122 10.1037/0096-1523.31.3.398

