# Supplementary material for: Comparison of walking overground and in a Computer Assisted Rehabilitation Environment (CAREN) in individuals with and without transtibial amputation
Source: J Neuroeng Rehabil. 2012 Nov 14;9:81. doi: 10.1186/1743-0003-9-81 (PMC3543217; doi:10.1186/1743-0003-9-81)
Supplement: Additional file 1 — Table S1. Mean and standard deviation of the temporal-spatial parameters during overground (OG) and treadmill walking in a CAREN (CA). Significant differences between conditions are highlighted. Significant p-values (p < 0.05) are in bold. [file 1743-0003-9-81-S1.pdf]

Supplemental Material

Table S1: Mean and standard deviation of the temporal-spatial parameters during overground (OG) and treadmill walking in a CAREN (CA). Significant differences between conditions are highlighted. Significant p-values ($p < 0.05$) are in bold.

	Controls						Patients with Transtibial Amputations								
	Combined Right & Left Sides			p-values			Intact			Prosthetic			p-values		
	OG Mean (SD)	CA Mean (SD)	Mean Diff.	Condition (OG/CA)	Side (R/L)	Side x Condition	OG Mean (SD)	CA Mean (SD)	Mean Diff.	OG Mean (SD)	CA Mean (SD)	Mean Diff.	Condition (OG/CA)	Side (I/P)	Side x Condition
Step Length (cm)	69.1 (5.1)	67.5 (5.1)	1.6	**0.005**	0.743	0.361	72.0 (4.2)	75.7 (3.8)	3.7	74.0 (3.8)	71.7 (3.5)	2.3	0.567	0.066	**0.016†**
Step Time (ms)	58.1 (3.1)	55.2 (2.8)	2.9	**0.000**	0.863	0.151	60.1 (1.8)	58.6 (1.7)	1.5	58.7 (1.9)	55.5 (2.2)	3.2	**0.032**	**0.046**	**0.035***
Step Width (cm)	10.9 (3.1)	11.6 (3.3)	0.7	0.082	0.005	0.520	11.7 (2.0)	14.9 (4.6)	3.2	11.9 (2.8)	14.4 (4.8)	2.5	0.110	0.762	0.383
Step Length Variability (cm)	1.6 (0.6)	1.8 (0.6)	0.2	0.132	0.601	0.106	1.6 (0.8)	2.7 (1.0)	1.7	1.9 (0.9)	1.5 (0.6)	0.4	0.052	**0.037**	0.066
Step Time Variability (ms)	1.2 (0.5)	1.1 (0.3)	0.1	0.479	0.967	0.313	1.5 (0.7)	1.2 (0.5)	0.3	1.1 (0.6)	1.0 (0.2)	0.1	0.423	**0.040**	0.527
Step Width Variability (cm)	1.9 (0.7)	2.3 (0.7)	0.4	**0.004**	0.794	0.269	2.8 (0.8)	3.6 (0.6)	0.8	2.5 (0.5)	3.4 (1.1)	0.9	0.029	0.349	0.940

† Significant difference between sides when walking in the CAREN. Overground and CAREN step length were different on the intact limb.

* Differences in step time between overground and CAREN walking were greater on the prosthetic side.

Table S2. The mean, standard deviation, and results of statistical comparisons are provided for various peak angles during the gait cycle. Data for pelvic and trunk angles are given for range of motion (ROM). Data provided for CAREN (CA) and overground (OG) trials are given in degrees. Significant differences between conditions (P< 0.05) are highlighted. Limbs are combined for mean and standard deviations (SD) provided. When available, minimum detectable change, MDC, values from a study of healthy, young individuals [1] are provided for comparison. Significant p-values (p < 0.05) are in bold.

	Controls						Patients with Transtibial amputations						
Kinematic Variable	OG Mean (SD)	CA Mean (SD)	Average Diff.	p-value Condition	p-value Side	p-value Side x Condition	OG Mean (SD)	CA Mean (SD)	Average Diff.	p-value Condition	p-value Side	p-value Side x Condition	MDC
Ankle Plantarflexion Peak, Early Stance	-5.2 (2.6)	-5.6 (2.8)	0.4	0.118	0.123	0.232	-3.3 (3.1)	-4.0 (3.4)	0.7	0.172	0.137	0.959	2.35
Ankle Dorsiflexion Peak, Late Stance	14.5 (3.2)	13.7 (3.2)	0.8	0.083	0.033	0.994	15.3 (2.0)	14.9 (2.1)	0.4	0.198	0.301	0.989	2.88
Ankle Swing Dorsiflexion Peak	2.6 (2.4)	1.7 (2.5)	0.9	**0.026**	0.145	0.417	5.5 (2.8)	5.1 (2.8)	0.4	0.494	0.118	0.386	*
Knee Flexion Peak, Early Stance	11.1 (5.5)	9.9 (5.3)	1.2	**0.040**	0.346	0.943	7.2 (3.2)	8.1 (4.3)	0.9	0.346	**0.001**	0.081	4.6
Knee Extension Peak, Late Stance	0.2 (4.1)	-0.7 (3.9)	0.9	**0.003**	0.760	0.876	-1.8 (3.4)	-3.1 (2.6)	1.3	**0.025**	0.234	0.835	4.7
Knee Flexion Peak, Swing	60.7 (3.7)	61.4 (3.8)	0.7	**0.043**	0.504	0.092	56.7 (5.7)	58.8 (5.4)	2.4	**0.042**	0.358	0.890	4.75
Hip Flexion Peak, Early Stance	27.4 (5.9)	26.4 (6.0)	1.0	0.053	0.503	0.272	23.4 (5.2)	25.5 (5.3)	2.1	0.063	**0.000**	0.718	1.36
Hip Extension Peak, Late Stance	-8.5 (5.9)	-8.8 (5.9)	0.3	0.385	0.623	0.325	-15.4 (3.8)	-15.2 (4.0)	0.2	0.846	0.103	0.620	1.35
Hip Flexion Peak, Swing	30.6 (6.2)	31.1 (6.2)	0.5	0.227	0.296	0.535	25.0 (5.4)	27.2 (5.5)	2.2	0.056	**0.003**	0.996	1.4
Pelvic Obliquity, ROM	14.2 (3.9)	11.1 (2.7)	3.1	**< 0.001**	0.075	0.761	5.0 (1.7)	5.2 (1.7)	0.2	0.729	**0.049**	0.812	2.52
Pelvic Rotation, ROM	15.3 (3.6)	12.6 (3.5)	2.7	**< 0.001**	0.161	0.466	8.8 (2.3)	8.3 (2.5)	0.5	0.438	0.408	0.802	2.21
Pelvic Tilt, ROM	3.6 (1.0)	3.3 (1.3)	0.3	0.084	0.320	0.399	3.5 (1.0)	3.6 (0.8)	0.1	0.807	0.648	0.662	0.64
Trunk Lateral Lean, ROM	5.5 (1.6)	3.7 (1.5)	1.8	**< 0.001**	0.513	0.890	7.0 (2.4)	7.2 (1.9)	0.2	0.605	0.149	0.297	1.13
Trunk Rotation, ROM	7.1 (2.3)	5.7 (1.9)	1.4	**0.004**	0.093	0.796	9.6 (2.4)	9.6 (2.9)	0.0	0.929	0.578	0.718	1.62
Trunk Anterior Lean, ROM	3.6 (0.8)	3.4 (0.7)	0.2	0.220	0.657	0.409	3.1 (0.5)	3.6 (0.7)	0.5	0.090	0.097	0.737	0.88

*Not provided in manuscript

1. Wilken, J., K. Rodriguez, M. Brawner, and B. Darter, **Reliability and Minimal Detectible Change Values for Gait Kinematics and Kinetics in Healthy Adults**. *Gait Posture*, In Press.

Table S3: Mean and standard deviation of kinematic variability, MeanSD, during overground (OG) and treadmill walking in a CAREN (CA). Significant differences between conditions are highlighted. Significant p-values ($p < 0.05$) are in bold.

	**Controls**						**Patients with Transtibial Amputations**								
	Combined Right & Left Sides			p-values			Intact			Prosthetic			p-values		
**MeanSD (deg)**	OG Mean (SD)	CA Mean (SD)	Mean Diff.	Condition (OG/CA)	Side (R/L)	Side x Condition	OG Mean (SD)	CA Mean (SD)	Mean Diff.	OG Mean (SD)	CA Mean (SD)	Mean Diff.	Condition (OG/CA)	Side (I/P)	Side x Condition
Ankle	1.05 (0.25)	1.07 (0.26)	0.02	0.543	0.064	0.793	1.03 (0.27)	1.10 (0.32)	0.07	0.44 (0.14)	0.35 (0.87)	0.09	0.872	**0.000**	**0.044†**
Knee	1.43 (0.30)	1.55 (0.30)	0.12	**0.031**	0.110	0.882	1.47 (0.32)	1.45 (0.30)	0.02	1.37 (0.40)	1.42 (0.52)	0.05	0.880	0.611	0.750
Hip	0.84 (0.19)	0.86 (0.18)	0.02	0.499	**0.017**	0.914	0.88 (0.16)	0.93 (0.13)	0.05	0.92 (0.19)	0.79 (0.22)	0.2	0.487	0.486	**0.035***
Pelvic Obliquity	0.85 (0.30)	0.70 (0.20)	0.14	0.410	0.843	0.065	0.43 (0.10)	0.47 (0.11)	0.04	0.42 (0.10)	0.48 (0.11)	0.06	0.162	0.949	0.726
Pelvic Rotation	0.74 (0.17)	0.77 (0.26)	0.08	0.092	0.859	0.298	1.18 (0.23)	1.17 (0.30)	0.01	1.22 (0.19)	1.17 (0.28)	0.05	0.837	0.634	0.612
Pelvic Anterior Tilt	0.87 (0.41)	0.76 (0.31)	0.11	0.201	0.847	0.118	0.65 (0.10)	0.68 (0.14)	0.03	0.71 (0.10)	0.68 (0.14)	0.03	0.963	0.302	0.286
Trunk Lateral Lean	0.64 (0.17)	0.60 (0.20)	0.04	0.382	0.906	0.630	0.67 (0.21)	0.86 (0.48)	0.19	0.58 (0.13)	0.86 (0.49)	0.28	0.149	**0.046**	0.057
Trunk Rotation	1.12 (0.41)	1.09 (0.23)	0.02	0.747	0.750	0.390	1.10 (0.21)	1.13 (0.33)	0.03	1.11 (0.18)	1.12 (0.32)	0.01	0.820	1.000	0.730
Trunk Anterior Lean	0.85 (0.43)	0.80 (0.31)	0.05	0.662	0.491	0.055	0.79 (0.15)	1.04 (0.24)	0.25	1.12 (0.33)	1.02 (0.23)	0.10	0.096	0.203	0.054

† No significant condition difference on either side

* Prosthetic side is less variable when patients walked in the CAREN
